# Energy Efficient UAV Flight Path Model for Cluster Head Selection in Next-Generation Wireless Sensor Networks

**DOI:** 10.3390/s21248445

**Published:** 2021-12-17

**Authors:** Syed Kamran Haider, Aimin Jiang, Ahmad Almogren, Ateeq Ur Rehman, Abbas Ahmed, Wali Ullah Khan, Habib Hamam

**Affiliations:** 1 College of Internet of Things (IoT) Engineering, Hohai University, Changzhou 213001, China; kamranhaider85@yahoo.com (S.K.H.); ateqrehman@gmail.com (A.U.R.); 2 Department of Electrical & Electronics Engineering, Beaconhouse International College, Islamabad 44000, Pakistan; abbas.ahmed@bic.edu.pk; 3 Department of Computer Science, College of Computer and Information Sciences, King Saud University, Riyadh 11633, Saudi Arabia; ahalmogren@ksu.edu.sa; 4 Department of Electrical Engineering, Government College University, Lahore 54000, Pakistan; 5 Interdisciplinary Centre for Security, Reliability and Trust (SnT), University of Luxembourg, 1855 Luxembourg, Luxembourg; waliullah.khan@uni.lu; 6 Faculty of Engineering, Uni de Moncton, Moncton, NB E1A 3E9, Canada; habib.hamam@umoncton.ca; 7 Spectrum of Knowledge Production & Skills Development, Sfax 3027, Tunisia; 8 Department of Electrical and Electronic Engineering Science, School of Electrical Engineering, University of Johannesburg, Johannesburg 2006, South Africa

**Keywords:** next-generation wireless sensor network, clustering, UAV flight path modeling, cluster balanced structure

## Abstract

Wireless sensor networks (WSNs) are one of the fundamental infrastructures for Internet of Things (IoTs) technology. Efficient energy consumption is one of the greatest challenges in WSNs because of its resource-constrained sensor nodes (SNs). Clustering techniques can significantly help resolve this issue and extend the network’s lifespan. In clustering, WSN is divided into various clusters, and a cluster head (CH) is selected in each cluster. The selection of appropriate CHs highly influences the clustering technique, and poor cluster structures lead toward the early death of WSNs. In this paper, we propose an energy-efficient clustering and cluster head selection technique for next-generation wireless sensor networks (NG-WSNs). The proposed clustering approach is based on the midpoint technique, considering residual energy and distance among nodes. It distributes the sensors uniformly creating balanced clusters, and uses multihop communication for distant CHs to the base station (BS). We consider a four-layer hierarchical network composed of SNs, CHs, unmanned aerial vehicle (UAV), and BS. The UAV brings the advantage of flexibility and mobility; it shortens the communication range of sensors, which leads to an extended lifetime. Finally, a simulated annealing algorithm is applied for the optimal trajectory of the UAV according to the ground sensor network. The experimental results show that the proposed approach outperforms with respect to energy efficiency and network lifetime when compared with state-of-the-art techniques from recent literature.

## 1. Introduction

The rapid growth and intensive development in the areas of wireless communication and computation science, including wireless sensor networks (WSNs) and other related technologies, is increasingly being used to satisfy evolving user requirements [1,2,3]. WSNs have increased flexibility in terms of maintenance and deployment when compared to conventional sensor networks. Due to the high demand and efficient scalability of WSNs, it has invaded numerous sectors. It has a prominent place in every corner of society, particularly in applications such as smart cities, industry 4.0, precise agriculture, and farming management [4,5,6]. WSNs have the attributes of significance and superiority and have been implemented in several domains due to increased flexibility and low cost. WSNs also play a pivotal role in environmental monitoring by gathering critical environmental parameters such as temperature, noise, fire detection, pollution, among many others. [7,8,9]. WSNs have seen substantial advancement in recent decades, particularly concerning data processing, communication quality improvements, energy saving, and data storage capacities. It has prompted the development in advanced technology domains of Cloud Computing, Big Data, and the Internet of Things.

In the standard architecture of WSN, its physical arrangement involves a large number of sensor nodes (SNs), each having a radio frequency (RF) transceiver system, intelligent microprocessor, storage, and battery. However, many challenges encountered by WSNs have been investigated and well researched, such as the limited storage capacity, energy constraints, and extensive deployment range required [10,11]. Besides the challenges mentioned above, particularly for applications such as monitoring and data gathering, two additional aspects need to be explored. First, a WSN comprises of static (fixed) placement of SNs. Although this static topology brings advantages of energy and cost efficiency, the overall system still lacks agility and mobility.

Furthermore, the static deployment of SNs restricts scalability and applicability. For environmental surveillance in large regions, there are inconsistencies between the increasing range of surveillance areas and the limitation of the surveillance scope of traditional WSNs. In a WSN monitoring environment, different obstacles may hinder the path of the wireless signals, such as huge tall buildings, walls, trees, human presence, and machines. However, these obstacles may significantly influence the quality of communication and wireless signal strength during signal propagation and cause deep fading of wireless signals, attenuation, and strong reflections from the objects. 

**Figure 1:**
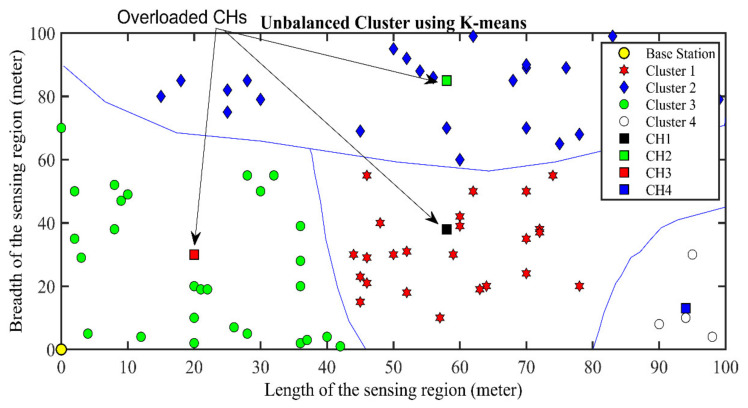
Unbalanced cluster formation by using K-means clustering technique.

Secondly, the clustering of WSNs is a critical aspect in many applications. Efficient clustering mechanisms can help achieve a longer life with energy conservation. In the clustering of nodes, the selection of cluster heads (CHs) and optimization of cluster structure are vital factors to be considered. The K-means clustering algorithm is widely used for cluster formation in different applications, including WSNs. However, K-means algorithms have certain drawbacks; such as the initial centroids are chosen randomly, leading to local optima, as seen in Figure 1. The figure shows there are four clusters in this simulation setup. Different colors represent the different clusters and their respective cluster heads. The cluster heads are denoted by a square mark, and separate shapes in each cluster represent the sensor nodes. There can be conditions where empty clusters or clusters having relatively low sensors are included. In cluster 4, there are only three sensor nodes. K-means algorithm does not guarantee its convergence into the best results. Even the optimal cluster’s density also cannot be decided and is given as an input by the user. 

To solve these two important research problems, researchers have proposed several solutions and methodologies. Many robots or mobile land vehicles described in literature reports are used in WSNs where the mobile robot/vehicle can act as a sink node, a relay node, and a base station. Unmanned air vehicles (UAVs) are the best among all mobile platforms and robots [12,13], widely employed in applications such as aerial photography, agriculture, and environmental monitoring. Safety, ease of operation, adaptability, and a broad monitoring range are UAVs’ significant characteristics. In the literature, it can be seen that UAVs are used to improve the quality of service (QoS) as well as expanding the overall monitoring area, including the collection of data from SNs and transmitting it forward to the base station. However, data collection, unbalanced cluster formations, and the flight path to visit each cluster inside the WSN still need investigation and performance consideration. Therefore, UAV-based WSNs need critical and efficient solutions. The clustering problem also needs an efficient algorithm that produces balanced clusters compared to K-means and includes an optimization technique for CH selection, keeping residual energy in consideration along with Euclidean distance. Therefore, this article provides an efficient and effective architectural layout of WSN incorporating intelligent UAV-based surveillance systems. In a UAV-based data collection system, we use UAV to help form balanced WSN clusters. This cluster formation helps in the energy conservation of WSNs, leading to a longer lifetime of nodes. UAV helps in the data exchange from the WSNs to the BS, reducing the communication range. 

The main contributions can be summarized as follows:

⮚ An energy efficient clustering protocol is presented to solve the issue of unbalanced cluster structure and optimizes the CHs selection process. A uniformly distributed cluster is obtained with almost equal number of SNs; the initial CHs are not chosen randomly in this algorithm; rather, midpoint strategy is used to address this problem. This technique also considers its communication with the UAV while selecting the CHs.
⮚ Considering the land WSN network, a UAV flight path is determined, which can collect data from every cluster of WSN optimally. The cluster head is placed at the center of the cluster and collects data, which are then passed to the UAV.
⮚ Our extensive simulations validate our proposed algorithm’s performance and show the performance in terms of lifetime, cluster design, and energy consumption.

The remainder of the paper is organized as follows. Section 2 covers the related work from the literature; Section 3 explains the system model; Section 4 describes the proposed methodology; and, finally, the simulation results are presented in Section 5.

## 2. Related Work

Researchers have explored the area of WSN for many decades under various limitations and constraints. Based on different stages and applications, the optimization schemes and utilized objectives were also different. The models for WSN are hierarchical and flat topology [14]. In the early phase of WSN practice, the majority of applications used the flat model. In this model, all network nodes share the same status, hardware specifications, and functions. Numerous algorithms and communication protocols have already been proposed for this topology. Furthermore, this model has adverse effects on the network management system. Moreover, SNs close to the base station (BS) might demand more energy to communicate with other network nodes via multihop, causing early discharge of the battery, leading to a dead node. As a result, the whole system network lifespan is reduced. Contrary to the flat model, hierarchical network design is based on a group of nodes functioning as sink nodes, group leader, and other ordinary nodes. Every node performs its duty, such as data collection and data transmission [15]. The authors in [16,17,18] evaluate the computation energy efficiency maximization schemes for the enhancement of WSNs. 

As the WSNs research area was explored extensively, the heterogeneous sensor network evolved from the hierarchical topology of WSNs. In [19], the authors proposed a clustering scheme to optimize the heterogeneous network using a genetic algorithm. Several recent approaches were evaluated, with their outcomes indicating that this method outperforms and extends the network’s lifetime. The authors in [20] presented the idea to reduce communication overhead by using the energy-aware clustering hierarchy protocol. For effective data collection and routing in WSNs, a multilevel hierarchical architecture was adopted. The proposed scheme simulation outcomes showed that it consumes the least amount of energy.

The authors in [21] introduced a new methodology of reclustering that improves overall system efficiency by appropriate task management of SNs. In another work, [22] proposed the constrained coverage (CC) technique, which considered K-neighbors for each cluster by using two virtual forces, but this method may cause the decrease of SN lifetime and low coverage area of the network. Furthermore, researchers in [23] developed virtual force-based clustering, but this technique may cause an unstable lifetime of WSN. 

Low-energy adaptive clustering hierarchy (LEACH) is a primary classical protocol, giving the idea of clustering in a WSN and introduces hierarchical transmitting of data [24]. The clustering technique transforms the WSN into groups or a hierarchy of clusters that gather the data from their surroundings and send it to its respective cluster head (CH). The optimal selection of CHs in a WSN cluster can maximize the communication range and prolong the network’s lifetime. In every round, the method randomly chooses CHs stochastically. Then, the nominated CH communicates with every non-CH node in the cluster to collect the sensed data. Election of the best CH is a critical task as variety of conditions are required to be fulfilled for selecting the optimal node in the whole cluster [25]. These conditions include factors such as residual energy, range, throughput, and mobility of each SN.

The LEACH algorithm extends the network lifespan compared to multihop and direct transmission but still has many drawbacks and limitations. The CH is selected on a random basis, which does not ensure an optimal solution and leads to improper distribution of SNs in each cluster, making it unbalanced. The nodes having lower residual energy levels are assigned the same priority as those with higher residual energy levels for CH nomination. Thus, when an SN of lower energy is nominated for CH responsibilities, its energy level will drop out in a shorter period, reducing network lifetime [26]. In [27], enhanced research work was introduced that exploits the LEACH algorithm to increase the energy efficiency of WSN. The authors in [28] proposed an optimized zone-based energy efficient protocol (OZEEP) for optimum CHs selection and improved the clustering by incorporating genetic fuzzy systems (GFS). One of the critical issues in clustering is optimizing the CHs selection and improving the cluster structure. The K-means method is highly effective in producing clusters for a myriad of IoT-based WSN applications. Various K-means-based techniques are discussed for efficient clustering [29,30,31,32,33,34].

However, this past research focuses only on WSNs, excluding the performance and applications of UAVs for data collection and surveillance purposes. Furthermore, these methods do not evaluate the topography and quality of wireless transmission during the design and position for surveillance systems. However, all these key factors must be taken into account for UAV-based WSNs. The authors in [35] proposed distributed and centralized K-means clustering technique. Although it is a good scheme for WSN clustering, the researchers only considered the distance parameter in its evaluation. With the growing development of UAV involvement in WSN, numerous literature reports exist for UAV-based WSNs. The studies can be further classified into optimal algorithms and applications. The first phase of UAV integration with WSN has been evoked in many domains, such as healthcare observations [36], monitoring of animals [37], data collection for greenhouse gases [38], and agriculture units [39]. To develop high-quality systems, the authors in [40,41] introduced new architecture of UAV-based WSN and evaluated certain applications. However, they mainly consider specific WSN types without focusing on UAVs and ground network systems. During the second phase, work done by the authors in [42,43,44] still face challenges in WSN overall energy conservation, adopting several techniques to optimize the problems related to routing, transporting protocols, and MAC in UAV-based WSNs. From the perspective of UAV, several studies identify flying control, path planning, and many other issues [45,46,47]. These studies exposed a new direction of research into UAV-based WSN and its applications. In addition, the approaches that we have stated focus solely on the challenges and conditions from a single perspective, i.e., either UAV or WSN, and do not examine the aerial mobile robots and ground network systems as a unified system. This substantially restricts their applicability and integration for many remote-based large-scale surveillance systems. In short, substantial use of these techniques cannot accomplish all the structural layout objectives of UAV-aided WSN for environmental monitoring. Moreover, in [48], the particle swarm optimization (PSO) approach was adopted to reduce UAV travel time, energy consumption, and bit error rate (BER). The ground WSN must be recurrent to choose the optimal CH during a single time slot or over multiple time slots. Furthermore, due to change in network topology resulting from the change of the CHs, UAV involvement helps to recalculate the flight path. This approach depends on ideal assumptions, which cannot be considered realistic scenarios, and further effort and work are required before data can be gathered in advance.

## 3. System Model

In this section, the system model and preliminary concepts of our work are discussed. A scenario is considered where several nodes are deployed in a random manner to collect the environmental parameters such as temperature, humidity, etc. The overall architecture of the monitoring system includes a UAV sink node, sensor nodes, cluster heads, and a remote base station. Each cluster has a cluster head, which receives the data from the sensors and then transfers it to the UAV, and acts as a sink node. The UAV further transmits these data to the remote base station. The land system computes the UAV’s flying trajectory once the geographical positions of CHs are obtained. The computation of UAV’s flight path parameters such as distance and time are considered. 

**Figure 2:**
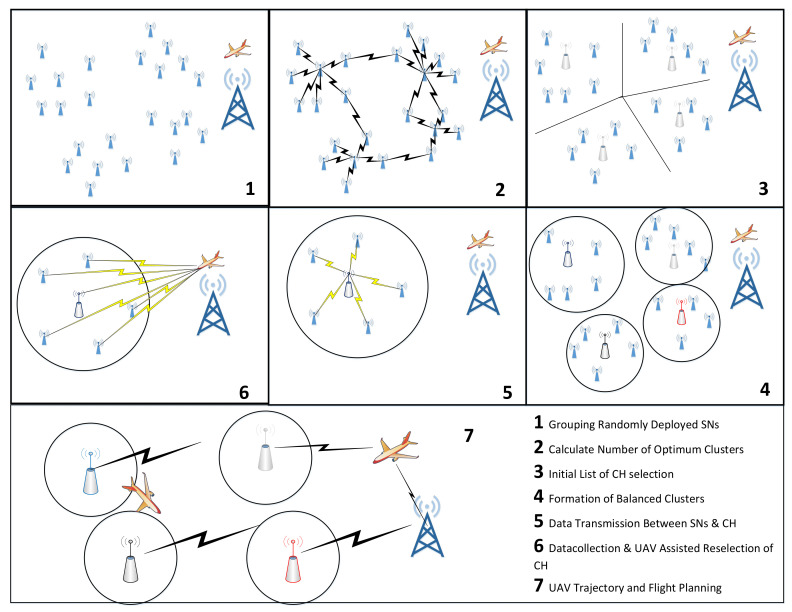
Stepwise contribution to the proposed method.

In the proposed system, the UAV is also utilized for the performance enhancement of the WSN, by making it more energy efficient in data collection and monitoring. In the proposed technique, optimized K-means clustering protocol is used to improve the cluster structure, CHs selection, and low-energy consumption for data communication. Figure 2 expresses the stepwise working of the proposed scheme, and the topology of the network considered. Table 1 gives the details of the symbols and notations.

In the scenario, a square range area with dimensions X×X is assumed, with N randomly deployed SNs in the sensing area. Both the SNs and the BS are static in nature, and there is only one UAV capable of flying over the sensing region. All the SNs are having same amount of initial energy and to be homogeneous in nature. The BS knows the geographical information of all deployed SNs. The proposed strategy starts with calculating the optimum cluster density in the area of interest, depending upon the sensing range and the total number of SNs. Mathematically, the number of optimum clusters can be calculated as follows [48]:

(1) $$C_{opt}=\left(\frac{\sqrt{N}}{\sqrt{2\pi }}\right)\left(\sqrt{\frac{\delta_{fs}}{\delta_{mp}}}\times \frac{X}{l_{BS}^{2}}\right)$$

where $l_{BS}$ is the distance between CH to BS, and $\delta_{fs}$ and $\delta_{mp}$ are parametric values for the free space and multipath model, respectively. The data are initially transferred to the BS, which shares this information with the UAV, to follow the CHs during its flight.

The next stage is to identify the cluster heads. Rather than picking the CHs by random means our proposed strategy uses midpoint technique. This methodology resolves the unbalanced cluster structure and uniformly deploys CHs to ensure that every cluster has almost equal SNs. This leads to an equal and balanced communication load on the CHs, which eventually expands the network’s life. This technique is explained in the next section. 

Our proposed technique considers residual energy of SNs along with the Euclidean distance for selection of CHs. The Euclidean distance is employed with the K-means basic approach; the nominated CHs transfer data to the UAV successfully. The K-means method is an iterative method that attempts to divide the dataset into K non-overlapping subgroups (clusters), where each element belongs to only one group. In the proposed scheme, K-means clustering categorizes the SNs into predefined C number of disjoint clusters. Algorithm 1 gives the idea of optimized K-means method.
**Algorithm 1:** Optimized K-means clustering method**Input:**X = consists of a total *n* number of data items.C = required clusters**Output:**A complete set of C clusters**Steps:**1: Choose C data items as initial centroids from X randomly.2: Repeat3: Associate each data item to the closest available centroid4: Mean value calculation for every cluster5: Continue until it meets the convergence criteria.

Another feature of our proposed clustering algorithm is that a node may not be nominated as CH if its remaining energy is less than a defined threshold. In our scenario, the estimate of the residual energy threshold is shown by the total energy needed for the aggregation, receiving and transmitting it to the average number of SNs in the cluster. Data aggregation happens in each of the selected CH and ultimately transferred to the UAV. 

This technique reduces the energy consumed by the CHs for data transfer. The communication range between CHs and UAV is kept small for data transfer. If the distance between CH and UAV is more than the threshold, the UAV will choose a different node, as CH is based on the Euclidean distance. Those nodes having good residual energy and better channel conditions will be considered in this process to improve the lifetime of the WSN. This mechanism can be called UAV-assisted re-election of CH, where the UAV performs the process under the TDMA scheme.

Once the clusters are formed and CHs are finalized, after determining CHs positions and geographical coordinates, the ground-based monitoring system computes the flight path for the UAV using an intelligent algorithm. UAV flies over CHs to function as sink node. It gathers all of the data and sends it to the base station for processing. The proposed flight trajectory for UAVs will visit each cluster for data collection from CHs in a shorter period and shorter path with the aim of low battery usage. Moreover, other aspects must be seen as the distance between the clusters, flight duration, and speed.

A radio energy dissipation model is used for performance evaluation of the proposed model. To transfer the k-bits message to a distance l, the radio utilized as follows:

(2) $$E_{transmit}\left(k,l\right)\;=\;E_{transmit-elec}\left(k\right)\;=\;E_{transmit-amp}\;\left(k,l\right)$$

(3) $$E_{transmit}\left(k,l\right)\;=\;E_{elec}\times \left(k\right)+\left(\delta_{fs}\times k\times l^{2}\right)\;\;\;\;\;\;\;\;if\;l<l_{0}$$

(4) $$E_{transmit}\left(k,l\right)\;=\;E_{elec}\times \left(k\right)+\left(\delta_{mp}\times k\times l^{4}\right)\;\;\;\;\;\;\;\;if\;l\geq l_{0}$$

where $E_{transmit-elec\;}\left(k\right)$ is transmit power by the electronic circuit to send 1-bit of data; $\delta_{fs}$ and $\delta_{mp}$ are the coefficients of free space and multipath models. In the free space model, their energy dissipations are proportional to $l^{2}$ for and in the case of multipath model proportional to $l^{4}$. However, the threshold $l_{0}$ is calculated as follows:

(5) $$l_{0}={\left(\frac{\delta_{fs}}{\delta_{mp}}\right)}^{\frac{1}{2}}$$

The energy required to receive the k-bits message at the receiving end is calculated as:

(6) $$E_{receive}\left(k\right)\;=\;E_{elec}\times k=E_{receive-elec}\left(k\right)$$

## 4. Proposed Method

The proposed energy efficient K-means protocol is explained in this section. As we know that energy efficiency is extremely important for WSN and UAV, our clustering approach reduces the energy consumption for both WSNs and UAVs. As explained previously, residual energy is considered in the clustering approach, which plays a vital role in CH selection. This optimized CH selection further influences the UAV by reducing the flight time, after designing the optimal trajectory for the UAV, hence significantly lowering its battery usage. The mathematical model of the proposed method is given in this section along with the pseudocodes in Algorithms 1–5.

### 4.1. Selection Strategy for the Initial Cluster Head

**Figure 3:**
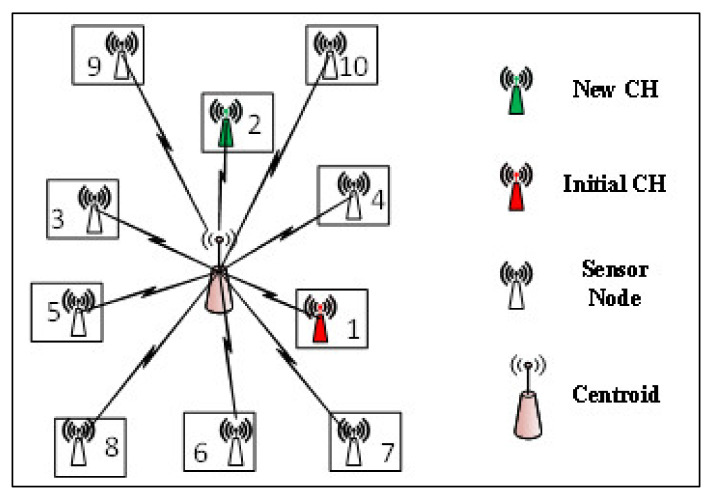
Midpoint point algorithm; IDs are based on the distances from the centroid.

In our proposed strategy, the midpoint method is used for initial CH selection by assuming only positive values for all selected data points n. The optimum cluster density Copt is obtained with the help of Equation (1). As shown in Figure 3, a total of ten SNs in a cluster are shown, where the midpoint method is applied to obtain the list of initial CHs. Here the centroid is a virtual node, positioning at the center of the cluster. In this figure, SN having ID number 1 and shown in red is initially elected CH. In every round, residual energy of the CH is observed to maintain the network connectivity and stability. If the current CH has residual energy lower than the threshold level, the next ID in the list is elected for new CH, which is 2, shown in green. The newly selected CH sends the beacon signal to all the nodes in a cluster for the change of CH. The working of this technique is shown in Algorithm 2.
**Algorithm 2:** Midpoint method for initial CH nomination**Input:**X = consists of a total *n* number of data points.C_opt_ = optimal cluster density**Output:**initial centroids of the C_opt_ clusters.**Steps:**1: Origin ($x_{o},y_{0}$), Data point *i* ($x_{i},y_{i}$)*For i = 1:n*$l\left(i\right)=\sqrt{{\left(x_{o}-x_{i}\right)}^{2}+{\left(y_{0}-y_{i}\right)}^{2}}$*end*2: Sort (l)3: *n*/C_opt_.4: The middle point value of each set is considered as the initial centroid.

**Algorithm 3:** Parametric approach for the balanced cluster structure
**Input:**
X = consists of a total *n* number of data items.C_opt_ = optimal clusters densityE_threshold_ = energy threshold
**Output:**
A complete set of C_opt_ clusters.
**Steps:**
1: Find C_opt_ initial CHs by using Algorithms 1 and 2.2: **Repeat**3: Rest of SNs join the nearest CH based on Euclidean distance.4: Centroid for each cluster:Centroid (x,y) = $\left(\frac{1}{S}\;\overset{S}{\underset{i=1}{\sum }}x_{i},\;\;\frac{1}{S}\;\overset{S}{\underset{i=1}{\sum }}y_{i}\right)$5: Once optimum cluster is formed, all SNs are assigned IDs based on the distance from centroid. Closer SNs will be assigned small numbers.6. For all selected CHs7:    **if** CH residual energy ≥ E_threshold_8:     **then**9:        CH won’t change10:   **else**11:       SNs ID numbers will be checked in the cluster12:       SN having next ID number is elected as a new CH.13: **End If**14: **End for**15: Beacon signal will be send to all SNs to inform them about the change of new CH.16: **Until** The CH residual energy meets the threshold level and no change in the CH anymore.

### 4.2. Methodology to Achieve Balanced Clusters

The balanced cluster structure phase is the next step in the process. The proposed approach includes a parameter of residual energy threshold for comparing the energy level of CH for each round. The threshold energy level is defined in terms of how much power it takes for each SN in the cluster to send, aggregate, and receive the average number of SNs. Hence, the threshold energy level is given by:

(7) $$E_{threshold\;}=\left(k\times E_{elec}\right)\times \left(\frac{N}{C_{opt}}-1\right)+\left(k\times E_{DA}\right)\times \left(\frac{N}{C_{opt}}\right)+\left(k\times E_{elec}\right)+\left(k\times \delta_{fs}\times d_{UAV}^{2}\right)$$

where *N* is the total number of SNs and Copt is the optimum cluster density. The detailed working of this step can be seen in Algorithm 3.

### 4.3. Energy Consumption of CH during Data Communication with WSNs and UAV Flight

The UAV spends most of its energy on flying, while some of its energy is utilized on collecting data from the CHs. On the other hand, the CHs spend energy on the data communication between CH and UAV and some on the communication within the cluster. Here the energy consumption based on our proposed clustering algorithm is analyzed and the approach used to reduce the UAV’s flight time by simulated annealing is also discussed.

#### 4.3.1. Energy Consumption in Proposed Clustering Approach

In Algorithm 4, the data communication model is proposed. As the distance range between the communicating CHs and the UAV is considered to be shorter than the threshold distance level of Equation (4), the model for free space radio energy given in Equation (2) is followed here. The set threshold level is 87.7 m for the communication of CHs and UAV. If CHs meet the threshold level, it can directly communicate with UAV, otherwise the nearest neighbor CH is used as the new nominated CH. After cluster formation, UAV calculates the number of SNs nsn for each cluster. The total energy of the CH, the distance of which for one round $l_{UAV}\;\leq \;l_{threshold}$, may be calculated as follows:

(8) $$E_{CH-R}=\left(k\times E_{elec}\right)\left(\left(n_{sn}-1\right)+\frac{c_{k}}{c_{opt}-c_{k}}\right)+\left(k\times E_{DA}\right)\left(n_{sn}+\frac{c_{k}}{c_{opt}-c_{k}}\right)+\left(k\times E_{elec}\right)+\left(k\times \delta_{fs}\times d_{UAV}^{2}\right)$$

where $n_{sn}$ represents the total SNs in that cluster, $c_{k}$ are the CHs unable to communicate or send data directly to the UAV, and $c_{opt}$ is the desired density of CHs. Hence, the value of $c_{k}$ ranges from 0 to ($c_{opt}-1$). For the non-CH member nodes the energy dissipation per round is:

(9) $$E_{n-CH}=\left(k\times E_{elec}\right)+\left(k\times \delta_{fs}\times d_{UAV}^{2}\right)$$

Our proposed method calculates the overall energy dissipation for a single round by using Equation (10):

(10) $$E_{rnd}=\underset{c_{opt}-c_{k}}{\sum }E_{CH-R}+\left(N-c_{opt}\;\right)E_{n-CH}\;$$

where *N* represents the total number of SNs distributed in the sensing field.
**Algorithm 4:** Modeling of Data Communication between CH and UAV**Input:**X = consists of a total *n* number of data items.{$CH_{1},\;CH_{2},\;CH_{3},\cdots \cdots ,\;CH_{C_{opt}}$} = A set of optimum Clusters, C_opt_*l*_threshold_ = distance threshold range = $\sqrt{\frac{\delta_{fs}}{\delta_{mp}}}=87.7\;m$**Steps:**1: CHs gets data packets from neighboring SNs.2: Compute the distance between each elected CH and UAV (*l*_UAV_)3: **If** (*l*_UAV_ < *l*_threshold_)4:   **then**5:     CH directly communicate to the UAV6: **else**7:     It selects the nearest neighbor CH whose *l* _UAV_ is less than *l*_threshold_ to communicate to the UAV.8: **End if**

#### 4.3.2. UAV Flight Planning by Using Simulation Annealing (SA) Approach

In this section we propose the UAV flight path planning methodology, which can minimize energy consumption and utilize the battery power sources effectively. The proposed method used the simulated annealing scheme to overcome the issues mentioned earlier. We can obtain the CH parameter vector as:

(11) $$X_{i}^{k}=\left\{X_{i1}^{k},X_{i2}^{k},X_{i3}^{k}\ldots \right\}\;$$

Moreover, the CH coordinates can be calculated as follows:

(12) $$C_{j}^{k}=\left\{\left(x_{1}^{k},\;\;\;\;\;y_{1}^{k}\right),\left(x_{2}^{k},\;\;\;\;\;y_{2}^{k}\right),\;\ldots ,\;\left(x_{j}^{k},\;\;\;\;\;y_{j}^{k}\right)\right\}$$

The UAV needs to analyze the all-CHs coordinates for data collection. We implement Equation (12) to compute the distance between two CHs (such as *a* and *b*) within the WSN cluster.

(13) $$Z_{ab}=\sqrt{{\left(x_{a}-x_{b}\right)}^{2}+{\left(y_{a}-y_{b}\right)}^{2}}\;$$

In the SA approach, Metropolis rules (13) are used to calculate the probability of acceptance p, analyzing the following equation:

(14) $$p=\left\{\begin{array}{c} \;\;\;\;e^{-\frac{dv}{T}}\;\;dv\geq 0\\ 1\;\;\;\;\;\;\;\;\;\;\;dv<0 \end{array}\right.$$

where T represents the current temperature, $dv=v\left(l^{m}\right)-v\left(l^{m+1}\right)$, and $v\left(l^{m}\right)$ is the path length for mth iteration. SA works with the key objective of identifying the shortest flight path $v_{min}\left(l\right)$ and the target points for flight sequence represented by $D^{v}$. The pseudocode of SA for UAV flight path planning is shown in Algorithm 5. In the algorithm, $t_{0}$ and $t_{F}$ are the initial and end temperature, respectively. At each interval, t is reduced step-by-step toward $t=\alpha t_{0}$, where α represents temperature decay factor.
**Algorithm 5:** Simulation Annealing Method for UAV flight path planning**Input:**CH coordinates $C_{j}^{k}=\left\{\left(x_{1}^{k},\;\;y_{1}^{k}\right),\;\;\;\left(x_{2}^{k},\;\;y_{2}^{k}\right),\;\ldots .,\;\;\left(x_{j}^{k},\;\;y_{j}^{k}\right)\right\}$, $t_{0}$, $t_{F}$**Output:**UAV flight route sequence target points $D^{v}$ and $v_{min}\left(l\right)$**Steps:**1: **while** ($t>t_{f}$)2:    create a new Hamiltonian circuit $l_{m+1}$ and calculate $v(l_{m+1})$3:    $dv\;\leftarrow \;v\left(l^{m}\right)-v\left(l^{m+1}\right)\;$4:    **if** (dv<0) // calculate probability of acceptance p5:      p ←16:    **Else**7:      $p\;\leftarrow \;e^{-\frac{dv}{T}}$8:    **End if**9:    **if** rand() ≤ p10:      update $D^{v}$ using $l_{m+1}$ and $v_{min}\left(l\right)=v\left(l_{m+1}\right)$11:    **Else**12:      discard $l_{m+1}$13:    **End if**14:    update the $t\;:t\;\leftarrow \alpha t_{0}$15: **End while**16: return $D^{v}$ and the $v_{min}\left(l\right)$

## 5. Simulation Results

To evaluate the performance of the proposed algorithm, simulations are conducted on MATLAB and the proposed approach is compared to similar studies from the literature. We consider two scenarios, one with dBS = 100, with the number of desired CH = 4, and the second with dBS = 85, which gets CH = 5. Each scenario has 100 SNs in the sensing field with dimensions 100 × 100 m^2^. Our proposed cluster formation technique is compared with Park’s approach [31]. The analysis also includes a comparison with existing approaches for different network parameters and characteristics such as energy consumption, number of living nodes, and the WSN’s data collection integrity. Table 2 shows the simulation parameters.

### 5.1. Cluster Structure Comparison

**Figure 4:**
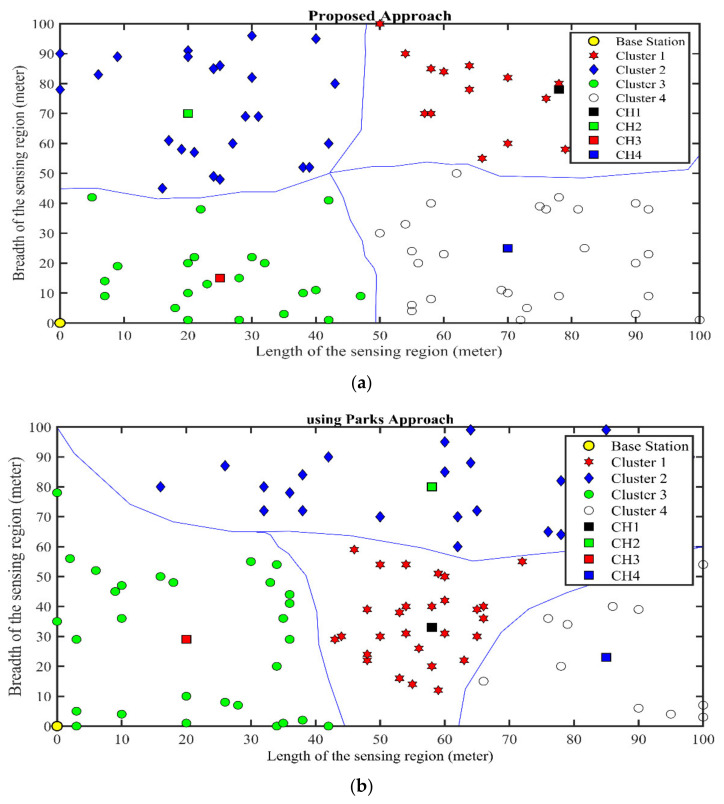
Midpoint point algorithm; IDs are based on the distances from the centroid. (**a**) K-means clustering approach, (**b**) Park’s clustering approach.

The proposed technique is applied for balanced cluster formation, with the midpoint algorithm for initial CH selection, as shown in Figure 4. It also shows the cluster structure after applying Park’s approach. After comparison, it is evident that there is a large variation in distribution of sensors between the clusters, while the proposed clustering approach has an almost equal distribution of nodes. Because of the unbalanced cluster structure, the CH with a high density of nodes will exhaust much earlier than the other clusters.

**Figure 5:**
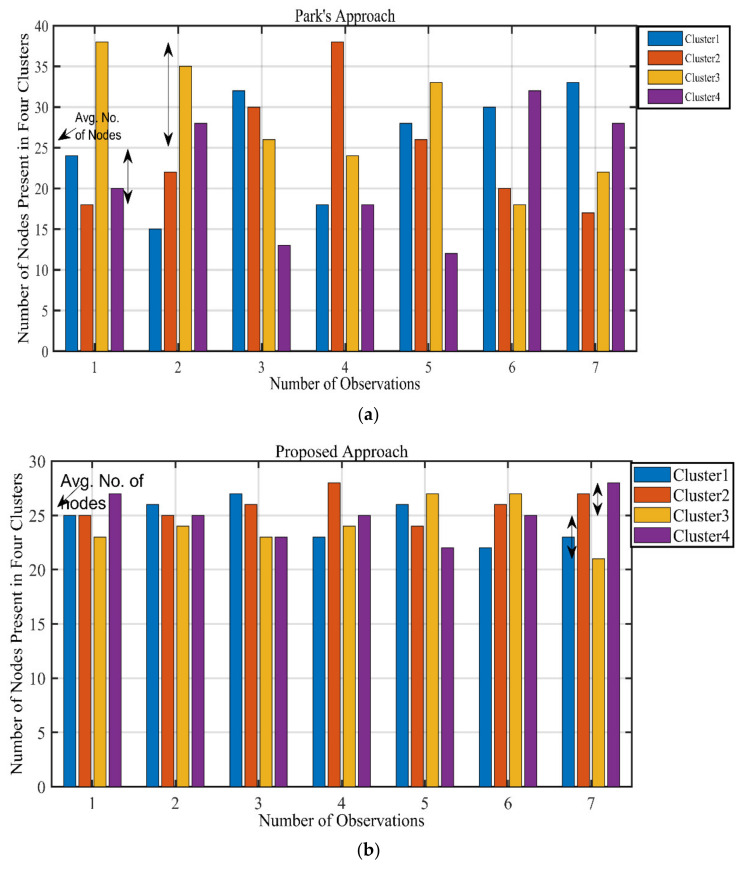
Clusterwise results for dBs = 100, 4 clusters. (**a**) Park’s approach, (**b**) Proposed approach.

To further validate our proposed clustering approach, we take seven observations for both dBs = 100 (4 clusters) and dBs = 85 (5 clusters). In the dBs = 100 scenario of 4 clusters the average number of SNs for each cluster is 25, the results when Park’s approach and our proposed approach are applied can be seen in Figure 5. A very clear difference can be seen, Park’s approach allocates the nodes in severely random way among the clusters; it can give as many as 38 nodes to a cluster and as low as 13 nodes to a cluster, which are both far from the ideal number of SNs. Alternatively, our proposed approach significantly reduces this window with the maximum of 28 SNs in cluster and minimum as 23 SNs. The results for dBs = 100 are also summarized in Table 3. 

**Figure 6:**
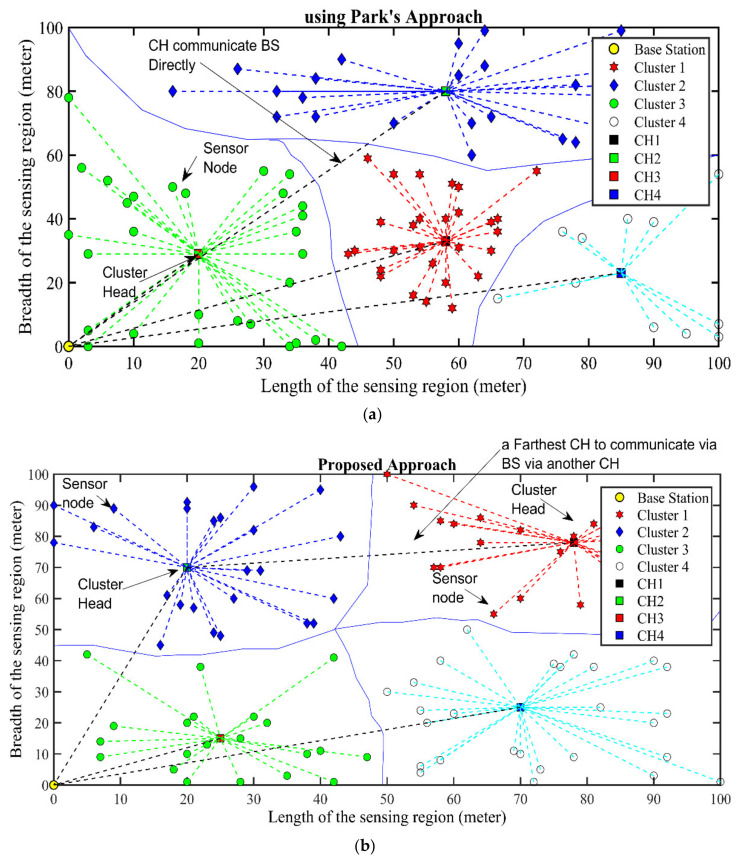
CHs to BS and ground-located UAV communication model. (**a**) Park’s approach, (**b**) Proposed approach.

We also analyze the proposed approach and Park’s approach from another point of view. In Park’s approach the distance between the CHs and BS initial position is not considered, and communication is performed in a single-hop manner without any involvement of UAV. This single-hop communication leads to high energy consumption for the CHs, which are at a large distance from the BS. Our proposed approach compares this distance between CH and ground-positioned UAV located at same position with BS, and if it is found greater than the threshold, then CH will communicate with the UAV via another CH in a multihop manner. Consequently, enhanced network lifetime is achieved. The simulation results can be seen in Figure 6.

**Figure 7:**
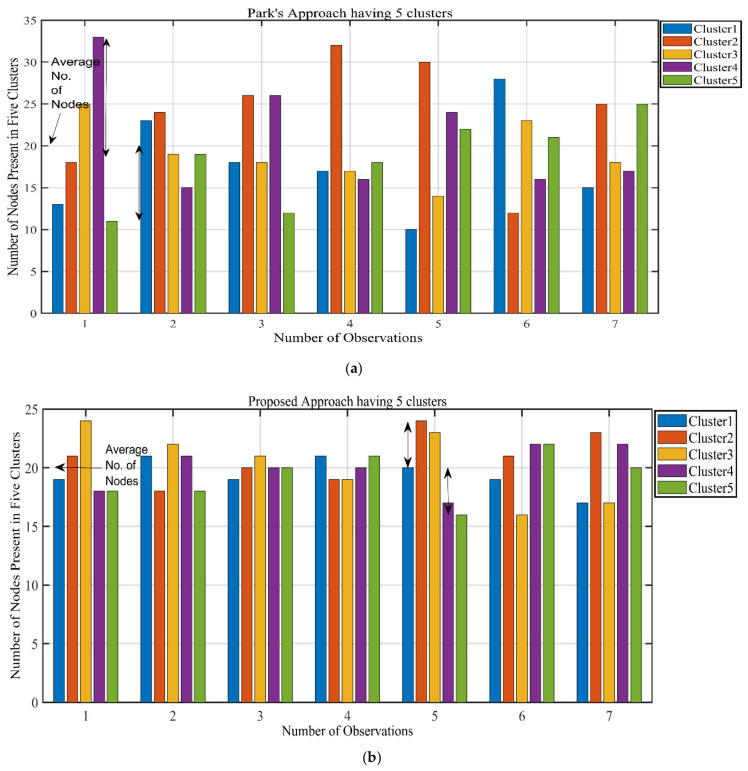
Clusterwise results for dBs = 85, 5 clusters. (**a**) Park’s approach, (**b**) Proposed approach.

We also analyze the dBs = 85 scenario, with 5 clusters and an average of 20 SNs per cluster. Figure 7 shows the results of applying the Park’s approach and proposed approach to this scenario. Similar to the dBs = 100 scenario, it can be seen how the proposed approach provides balanced clusters. Park’s approach can give as high as 33 nodes to a cluster and as low as 10 nodes to a cluster, while the average is 20 nodes per cluster. Our proposed approach achieves a balanced cluster scenario, with the highest number of nodes in a cluster as 24 and the lowest number of nodes as 16. The results for dBs = 85 are also summarized in Table 4.

Our clustering approach is closer to the ideal cluster structure, which can be seen by using the standard deviation parameter. For a set of *n* numbers *x*_1_, *x*_2_, *x*_3_, …, *x_n_* it can be mathematically given as follows:

(15) $$\mathrm{Standard}\;\mathrm{deviation}\;\left(\sigma \right)=\sqrt{\frac{1}{n}\sum {\left(x_{i}-\bar{x}\right)}^{2}}$$

The analysis is done using Equation (15) and the data from Table 3 and Table 4. Since the area has a total of 100 sensors, it makes 25 nodes per cluster in the 4-cluster scenario and 20 nodes per cluster in the 5-cluster scenario as the ideal count. Table 5 shows the details for measure of dispersion in both the 4- and 5-cluster scenarios. Park’s approach shows a greater dispersion as compared to our proposed approach. It clearly makes our approach as the more suitable choice, as it returns balanced clusters leading to better network lifetime. 

### 5.2. Network Lifetime Comparison

**Figure 8:**
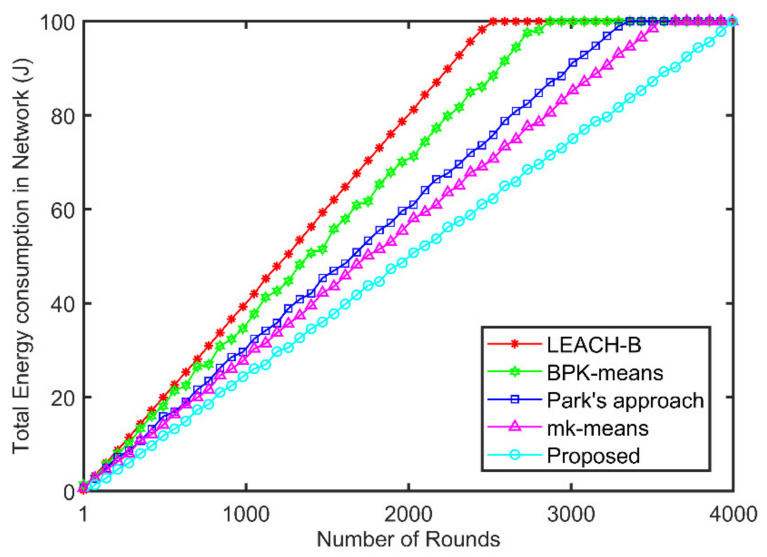
Network lifetime comparison.

The network lifetime comparisons of our method are made with four conventional methods namely, LEACH-B, BPK-means, Park’s approach, and mk-means. Figure 8 shows the comparison based on network lifetime and the proposed method shows a higher lifetime compared to the other techniques. The number of live nodes reported against each round, the group leader selection criteria, and clustering approach makes our methodology more robust. The results are summarized in Table 6.

### 5.3. Energy Efficiency Comparison

**Figure 9:**
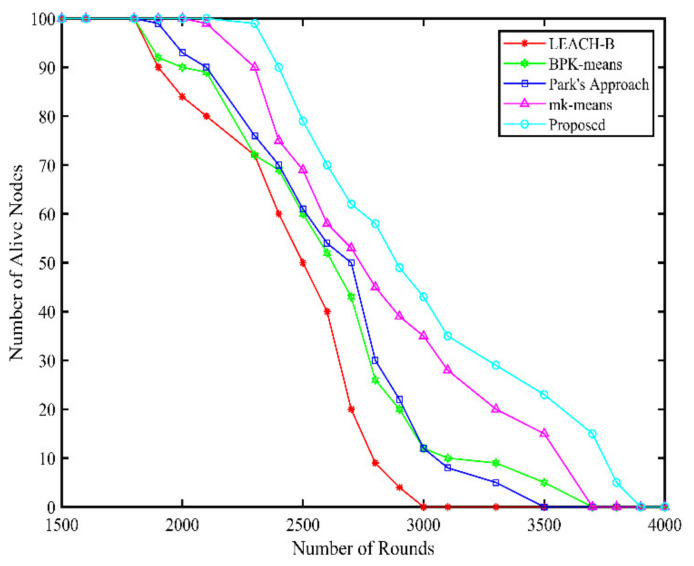
Energy consumption per round.

Energy consumption comparison of our proposed method with LEACH-B, BPK-means, Park’s approach, and mk-means algorithm is made for the number of rounds. Figure 9 shows that the proposed method can significantly reduce energy consumption compared to the other four algorithms. The detailed analysis of this figure is given in Table 7. In order to calculate the network lifetime, the definition of one round is given in our previous work [32].

In addition to the analysis given above, the summary of our proposed work novelty and contribution are summarized in Table 8.

### 5.4. UAV Flight Path

**Figure 10:**
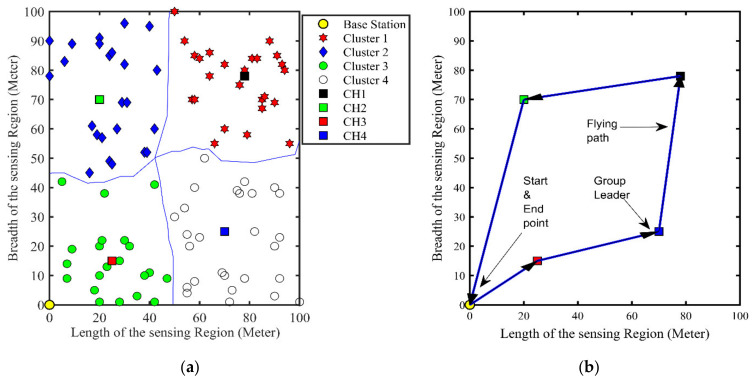
UAV flight trajectory. (**a**) Nominated cluster head for communication, (**b**) UAV flying route for data gathering.

The proposed clustering algorithm resolves the balance cluster problem of land-WSNs, by creating distance and residual energy-based clusters. The UAV trajectory to cover all CHs is also provided. The SNs transfer their data to the CH and the UAV flying over the CHs collects the data. In Figure 10a part shows four clusters along with their nominated CHs. In Figure 10b, part of the blue line represents the flight path, starting from the base station, passing through each CH from all the clusters and returning to its initial position.

## 6. Conclusions

In this work, a clustering approach for WSNs is proposed, which aims to reduce the energy consumption and extend the network lifetime. The proposed approach effectively groups the SNs into balanced clusters by merging midpoint technique with the K-means clustering algorithm. Instead of random initial centroids, a systematic method is adopted in our technique. The optimization approach takes the residual energy along with Euclidean distance and position of SNs. Multihop communication between the CHs to deliver data to the UAV limits the energy consumption of nodes. A classical method is used to shape the optimal flight trajectory of the UAV to collect the data from the CHs. Our simulations clearly indicate the superiority of our proposed methodology as compared to the LEACH-B, BPK-means, mk-means, and Parks approach, with percentages of 50%, 14%, 10% and 6%, respectively. In future work, we may consider the factor of reusability for CHs by adapting machine learning algorithms and also consider the optimized UAV flight energy path loss. 

## Figures and Tables

**Table 1:** Symbols and notations.

Symbol/Notation	Details
$l_{BS}$	Distance between CH to BS
$\delta_{fs}$	Parametric values for the free space
$\delta_{mp}$	Parametric values for the multipath
$C_{opt}$	Optimal number of clusters
X	Side length of the sensing area
N	Number of SNs in the sensing area
k	Message length in bits
*l*	Distance for transmitting k bits
$l_{0}$	Threshold
$E_{transmit-elec}\left(k\right)$	Transmit power by the electronic circuit to send k-bit of data
$E_{receive}\left(k\right)$	Energy required to receive the *k*-bits message at the receiving end
$E_{threshold}$	Threshold energy level
$n_{sn}$	Total SNs in the cluster
$c_{k}$	CHs unable to communicate or send data directly to the UAV
*l*_UAV_	Distance between each elected CH and UAV
dv	Current temperature
$Z_{ab}$	Distance between two cluster heads

**Table 2:** Simulation parameters.

Parameter	Value	Unit
network size	100 × 100	m^2^
base station location	(0, 0)	
number of clusters (*C_opt_*)	4, 5	
number of sensor nodes (N)	100	
$E_{elec}$	50	nJ/bit
$\delta_{mp}$	0.0013	pJ/bit/m^4^
$\delta_{fs}$	10	pJ/bit/m^2^
$\mathrm{energy}\;\mathrm{for}\;\mathrm{data}\;\mathrm{aggregation}\;(E_{DA}$)	5	nJ/bit/signal
initial energy of node	1	Joule
data packet	3200	bits
$l_{BS}$	85–100	m
D_threshold_	88	m
D_ICH_	$l_{BS}/$ ^2^	

**Table 3:** Balanced cluster comparison (dBs = 100).

	Cluster 1		Cluster 2		Cluster 3		Cluster 4	
Obs.	Park’s	Proposed	Park’s	Proposed	Park’s	Proposed	Park’s	Proposed
1	24	25	18	25	38	23	20	27
2	15	26	22	25	35	24	28	25
3	32	27	30	26	26	23	12	23
4	17	23	38	28	24	24	18	25
5	28	26	26	24	33	27	13	23
6	30	23	20	26	18	27	32	25
7	33	23	17	27	22	22	28	28

**Table 4:** Balanced cluster comparison (dBs = 85).

	Cluster 1		Cluster 2		Cluster 3		Cluster 4		Cluster 5	
Obs.	Park	Proposed	Park	Proposed	Park	Proposed	Park	Proposed	Park	Proposed
1	13	19	17	21	25	24	33	17	12	17
2	23	21	24	17	19	22	15	21	19	17
3	18	19	26	20	18	21	26	20	12	20
4	17	21	32	18	17	18	16	20	18	21
5	10	20	30	24	14	23	24	17	22	16
6	28	19	12	21	23	16	16	22	21	22
7	15	17	25	23	18	17	17	22	25	20

**Table 5:** Standard deviation from ideal cluster size.

	4-Clusters		5-Clusters	
Obs.	Park’s	Proposed	Park’s	Proposed
1	1.55	0.281	1.80	0.510
2	1.49	0.142	0.722	0.373
3	1.435	0.425	1.3	0.142
4	1.67	0.373	1.34	0.199
5	1.68	0.448	1.5	0.706
6	1.21	0.373	1.242	0.510
7	1.22	0.635	0.937	0.509
**Average**	1.465	0.382	1.264	0.421

**Table 6:** Network lifetime comparison detailed analysis.

Algorithm	Round 1st Node Dies	Round Half Nodes Dies	Round Last Node Dies
Proposed	2450	3080	3700
Mk-means	2210	2790	3570
Park’s approach	2200	2750	3400
BPK-means	2100	2700	3500
LEACH-B	1900	2350	2950

**Table 7:** Network lifetime comparison.

Algorithm	Number of Rounds
LEACH-B	1800
BPK-means	1850
Park’s approach	2050
mk-means	2200
Proposed	2400

**Table 8:** Comparison and summary of existing methods with our proposed method.

Key Features	Mk-Means	BPK-Means	Park’s Approach	Proposed Method
Based on	K-means method	K-means method	K-means method	Improved K-means with midpoint method approach
Initial selection of CHs	Randomly	Randomly	Randomly	Midpoint approach is used
Create balanced cluster structure	Yes	Yes	No	Yes
Compute optimum list of CHs	No	Yes	No	Yes
Clustering considers minimal distance between the SN and CH	No	Yes	Yes	Yes
residual energy taken into account for the selection of CH	Yes	No	Yes	Yes
Specified CH residual energy threshold level	Yes	Yes	No	Yes
Uniformly distribution of CHs over the sensing region	No	No	No	Yes
Supports multihop communication between the CH and the UAV	No	No	No	Yes
prolong network lifetime	Yes	No	Yes	Yes
